# Empathy is not so perfect! -For a descriptive and wide conception of empathy

**DOI:** 10.1007/s11019-022-10124-w

**Published:** 2022-11-15

**Authors:** Elodie Malbois, S. Hurst-Majno

**Affiliations:** Institute for Ethics, History, and the Humanities, University of Geneva, Geneva, Switzerland

**Keywords:** Empathy, Physician, Empathic concern, Medical education, Compassion, Empathic distress

## Abstract

Physician empathy is considered essential for good clinical care. Empirical evidence shows that it correlates with better patient satisfaction, compliance, and clinical outcomes. These data have nevertheless been criticized because of a lack of consistency and reliability. In this paper, we claim that these issues partly stem from the widespread idealization of empathy: we mistakenly assume that physician empathy always contributes to good care. This has prevented us from agreeing on a definition of empathy, from understanding the effects of its different components and from exploring its limits. This is problematic because physicians’ ignorance of the risks of empathy and of strategies to manage them can impact their work and wellbeing negatively. To address this problem, we explore the effects of the potential components of empathy and argue that it should be conceived as a purely descriptive and wide term. We end by discussing implications for medical education.

## Introduction

Physician empathy is considered essential for good care. Most often understood as understanding the patient and communicating that understanding to them (Hojat et al. 2001), physician empathy is important for patients to feel understood and cared for, which impacts their care. Researchers have therefore been looking for ways to enhance physician empathy. However, there are also methodological weaknesses in this literature, especially in the way empathy is defined and tested.

In this paper, we show how the idealization of physician empathy has been an underlying issue in that literature. Physician empathy has been approached with the mistaken assumption that it has only beneficial effects on care. This has prevented researchers from agreeing on a definition for empathy, from achieving a precise understanding of its effects and from understanding its limits. This is problematic because it prevents physicians from knowing about the risks of empathy and from learning how to manage them. We explore the effects of the potential components of empathy that have been identified in the cognitive sciences and conclude that we should adopt a descriptive definition of empathy that encompasses all the potential components.

Our line of argument goes as follows: we start by describing the state of understanding of the effects of empathy in medicine and explain that it is difficult to draw general conclusions from this literature because of methodological weaknesses. One major challenge is the variety of definitions of empathy being used. We then show (part 2) that one of the reasons why we cannot reach a consensus on how to define empathy is that we have idealized empathy and assumed that its effects on care were always positive. This assumption is also responsible for the lack of a precise understanding of the effects of the components of empathy and of the limits of their benefits in care. We proceed to fill that gap and describe the potential components of empathy based on the cognitive sciences (part 3) before exploring their effects in medicine (part 4). Based on this description, we argue that we should adopt a purely descriptive and all-encompassing definition of empathy. We end by outlining how this description can help us identify ways to teach physicians how to use empathy for sustainable good care (part 5).

### Physician empathy

Patients want their caretakers to be interested in them and to care about them (Bensing et al. 2013; Cheraghi‐Sohi et al. 2006; Vedsted and Heje 2008; Wensing et al. 1998). Physician empathy appears to greatly contribute to that. There is no consensus on how to define physician empathy, but most often, it is understood as the ability to cognitively understand the perspective and the emotions of a patient, the communication of that understanding to them and the motivation to help them (Mercer and Reynolds 2002; Pedersen 2009; Hojat et al. 2001; Del Canale et al. 2012; Kelm et al. 2014).

The importance of empathy in clinical care goes beyond patient satisfaction (Hojat et al. 2010). Feeling understood contributes to inspiring trust in the physician (Huntington and Kuhn 2003; Levinson et al. 2000; Pollak et al. 2011; Riess et al. 2012; Zachariae et al. 2003), which leads to better compliance and better healing outcomes (Del Canale et al. 2012; Hojat et al. 2011; Kim et al. 2004; Steinhausen et al. 2014; Sultan et al. 2011). A study by Del Canale et al. found that patients with diabetes that had physicians who scored high on empathy had a significantly lower risk of complications (Del Canale et al. 2012). Rakel and al. found that patients’ perception of physician empathy significantly predicted the duration and the severity of the common cold (Rakel et al. 2009). A review also concluded that perception of empathy directly correlates with the strengthening of patient enablement and that empathy “lowers patients’ anxiety and distress and delivers significantly better clinical outcomes” (Derksen et al. 2013). Furthermore, evidence suggests that patients who trust their physician and have a good doctor-patient relationship—for which empathy is instrumental—do not sue their physician (Hojat et al. 2002a, b; Levinson 1994; Huntington and Kuhn 2003). Finally, empathy seems to increase physicians’ health and wellbeing (Gleichgerrcht and Decety 2014; 2013). Since there is evidence, although disputed, that empathy in medicine students decreases with years of study (Colliver et al. 2010; Neumann et al. 2011),^1^ training programs are being developed to enhance students’ empathy, including with fiction and narrative medicine (Chen et al. 2017; Reilly et al. 2012; Riess et al. 2012; Shapiro et al. 2006).

While empirical data strongly suggests that empathy positively impacts care, it is difficult to draw general conclusions regarding the role of empathy in medicine because there is no agreement on how to define it (Decety 2020). The main disagreement is on the inclusion of an affective component. Although in its common acceptance, empathy is understood mainly as an emotional attitude, the understanding of the patient involved in physician empathy has traditionally been conceived as primarily cognitive (Hojat et al. 2011; Del Canale et al. 2012; Kelm et al. 2014). But some consider that an affective dimension such as feeling the patient’s emotions or feeling concern for them is central to physician empathy (Chen et al. 2017; Ekman and Krasner 2017; Patel et al. 2019; Roche and Harmon 2017; Guidi and Traversa 2021). In a review of research on empathy and medical education, Sulzer et al. found that 85% of the studies defined empathy as involving a cognitive component and 37% defined empathy as involving a “feeling process” (Sulzer et al. 2016). A behavioral component is also sometimes included (Sulzer et al. 2016; Pedersen 2009). Furthermore, many studies on empathy do not provide clear definitions of empathy at all (Decety 2020; Derksen et al. 2013; Pedersen 2009). In their review Sulzer et al. found that 20% of the studies selected “failed to define the central construct of empathy” (Sulzer et al. 2016).

In addition, there are issues with measures of empathy. Hemmerdinger et al. reviewed tests of empathy and found that out of 36 tests of empathy, only eight had evidence of reliability and validity (Hemmerdinger et al. 2007). The level of empathy measured also depended on the method used for more than half the studies Pedersen reviewed (Pedersen 2009). Most tests of empathy use self-reports that are usually filled in removed from any particular interaction with patients (Colliver et al. 2010; Pedersen 2009). The ability of those tests to predict a physician’s empathy is therefore unclear (Sulzer et al. 2016). Lastly, what is measured is not always empathy as defined in those studies. In their review, Sulzer and al. found that only 13% of the studies “used an operationalization that was well matched to the definition provided” (Sulzer et al. 2016).

Hence, studies on physician empathy do not all investigate the same phenomenon and use measures that are not always reliable. It is therefore difficult to draw general conclusions regarding the effects of empathy in medicine.

### The idealization of empathy and its effects

#### The idealization of empathy

The lack of agreement on how to define and measure empathy partly stems from the fact that empathy has been idealized. Empathy is a “thick concept”^2^ (Prinz 2011) which means that it has a descriptive and an evaluative component (Väyrynen 2021). For instance, when we say that Melinda’s action was generous, we both describe her action (she gave something she did not have to give willingly and happily) and evaluate it (she did good). Similarly, when we say that Albert was empathic to Nola last night, we say something about Albert’s state of mind at that time, and we make an evaluative judgement. Hence, it would be odd to say that Albert was empathic to Nola and that this was not good in any way (Väyrynen 2021). Despite some efforts to show that empathy is not necessarily good (Prinz 2011; Bloom 2017), the concept of empathy usually involves a positive evaluative component, especially in its common acceptance.

There are discussions on how the descriptive and normative components of thick concepts are related. Some argue that the descriptive conditions can be sufficient satisfaction conditions for the evaluative concept, while others disagree (Väyrynen 2021; Blomberg 2010). For instance, some might say that if one tells the truth, then necessarily one is honest and therefore good.^3^ Theories of physician empathy seem to have implicitly adhered to this view. It has been assumed that what satisfies the descriptive condition(s) of empathy amounts to empathy and is necessarily good. But since researchers disagree on what attitude of the physician is beneficial to care, they have been arguing on the descriptive conditions of empathy.

Physician empathy was initially conceived as mainly cognitive because being emotional was considered dangerous for physicians. In a seminal paper published in 1958, Charles Aring explained that the emotional attitude of sympathy, defined as “the act or capacity of entering into or sharing the feelings of another,” reduces the physician’s “freedom of movement” by making them react emotionally towards their patient. This prevents them from acting objectively with only the good of the patient in view (Aring 1958). Aring therefore encouraged physicians to rather have empathy towards their patients. He defined empathy as a “feeling-into” which sustains the “awareness of one's separateness from the observed” (Aring 1958). It is an intellectualized appreciation of another’s experience based on our own past experiences which does not require joining the patient’s experiences and feelings. A physician who is empathic rather than sympathetic towards patients “remain[s] unencumbered by the patient[s’] problem” which is “a subtle and significant feature of a happy medical practice” and enables them to be most effective (Aring 1958). Hence, emotional involvement was to be avoided and empathy was understood as a form of “detached concern” (Halpern 2003).

This worry that an emotional involvement with patients might impair physicians' judgments and make them more prone to burnout persisted throughout the twentieth century (Halpern 2003). However, there were also complaints in the late 1960s and 1970s that physicians were too insensitive and that their practice was dehumanizing (Halpern 2003). As a result, the ideal of detached concern started to fade away and the conception of empathy changed. Although it is still most often conceived as primarily cognitive (Hojat 2016; Del Canale et al. 2012; Kelm et al. 2014), it is now seen as a way to avoid indifference for what patients experience. The empathic physician is not the one who is emotionally detached, but the one who is sensitive and perceptive to their patients’ experiences, feelings, and values.

Some like Halpern go further and claim that emotional involvement with patients can be highly valuable and is therefore part of empathy (Halpern 2001). Being attuned to the patient’s emotions can make patients feel cared for and help the doctor-patient relationship and can help physicians understand their patients. Halpern provides an example from her own experience. After meeting with a patient who was a “successful executive... paralyzed from the neck down” and “ventilator-dependent” (Halpern 2001, p 86) who refused therapy, she “felt hopeless about returning to talk with him and thought that this reflected [her] own lack of clinical experience” (Halpern 2001, p 87). But someone else pointed that the hopelessness she was feeling might be her patient’s. This helped her to distinguish her feelings from her patient’s and enabled her to find the right way to approach him and to make him feel understood. Others have joined Halpern with her more emotional conception of physician empathy (Chen et al. 2017; Ekman and Krasner 2017; Patel et al. 2019; Roche and Harmon 2017).

Definitions of empathy tend to also include other features considered essential to positively impact care. For example, for patients to feel understood, physicians need to communicate their understanding of the patient to them. Therefore, communication of understanding is often involved in definitions of physician empathy (Roche and Harmon 2017; Hojat et al. 2018; Aomatsu et al. 2013; Derksen et al. 2013). A behavioral component such as a motivation to help is also frequently added on similar grounds (Hojat et al. 2018; Derksen et al. 2013).^4^

Hence, the widespread idealization of empathy implies that there cannot be a consensus on a definition unless we agree that a certain attitude of the physician is always good for care. Furthermore, this assumption also has other consequences.

#### The effects of empathy

One consequence is that we do not have a precise understanding of the effects of the different potential components of empathy. Most empirical studies are not intended to explore the effects of the components of empathy but rather to confirm that a certain concept of empathy has positive effects (Del Canale et al. 2012; Steinhausen et al. 2014; Rakel et al. 2009; Chen et al. 2017; Pollak et al. 2011).^5^ Many tests of empathy have been designed to provide one global score despite measuring different aspects of empathy. This is the case of the Jefferson Scale of Physician Empathy (Hojat et al. 2001), the Consultation and Relational Empathy (CARE) (Mercer and Reynolds 2002) and the Hogan’s empathy scale, which are among the most used ones.^6^ Sulzer et al. also found that two thirds of the studies they reviewed “operationalized empathy solely as a global construct that increases or decreases monolithically” (Sulzer et al. 2016).

As a result, we do not have a good understanding of the role that each of the potential components of empathy plays. For example, two physicians might have similar scores of empathy although one could be very skilled at taking their patients’ perspective and understanding them, but poor at communicating it, while the other could be a mediocre perspective-taker but care a lot about their patients. Those two ways of being empathic will impact care very differently, but empathy scores do not account for that. Furthermore, as described above, the number of tests used for empirical studies and issues with operationalization are further obstacles to drawing general conclusions from these data.

This implies that at the moment we cannot rely on empirical data to determine which potential components of empathy are beneficial to care and find a consensus on how to define empathy. Even the widespread idea that sharing the patient’s emotion is dangerous and causes burnout has not been verified. In a review, Wilkinson et al. found that both affective and cognitive empathy are negatively correlated with burnout (Wilkinson et al. 2017). While low perspective-taking alone might increase the risk of burnout (Lee et al. 2003; Lamothe et al. 2014), higher perspective-taking might be protective when associated with higher empathic concern—feelings of concern, sadness, worry, etc. for patients. A study by Gleichgerrcht and Decety also found that empathic concern was strongly associated with compassion satisfaction (Gleichgerrcht and Decety 2013). This suggests that emotional involvement from the physician is not necessarily harmful and might in fact be beneficial for the physicians themselves.

#### The limits of empathy

Another repercussion of the idealization of empathy is that its limits are rarely explored. A few researchers have voiced concerns regarding physician empathy. Garden argued that given the doctor-patient power differential, empathy can lead physicians to take themselves as authorities on what patient experience (Garden 2007). Smajdor, Stöckl and Satler also showed how empathy is not necessary nor sufficient for good medical practice and claim that courtesy might be more important (Smajdor et al. 2011). Among those who have studied empathy empirically, however, very few discuss what could possibly go wrong with physician empathy. Hojat et al. claim, for example, that “empathy has no restraining boundary because it is assumed that understanding is always beneficial in patient care. An abundance of empathy should never impede patient care” (Hojat et al. 2002a, b).

However, a physician might satisfy the descriptive component of empathy (however we conceive of it) and it might not produce any benefit or worse, it might impede care. They might have “the ability to understand another person’s inner experiences and feelings and a capability to view the outside world from the other person’s perspective” (Hojat et al. 2002a, b) and still fail to understand a patient. We are not as good as we think at perspective taking (Maibom 2018) and we can easily fail to appreciate how someone’s values, beliefs and experiences can differ from ours and how that impacts our perspective on the situation. We can also be unaware of important facts about another’s situation despite our best efforts to understand that person, such as past trauma that the patient hides.

Similarly, communication can be difficult and lead to misunderstandings. Even the motivation to help can have negative consequences such as overtreating. Furthermore, there are times when empathy might not be needed, such as during clinical reasoning or surgery (Smajdor et al. 2011). In addition, we know that empathy has a dark side: our ability to empathize is biased—we are more empathic towards in-group members—and empathy can motivate behavior that helps the target of empathy but is unfair towards others (Stürmer et al. 2006; Batson et al. 1995).

Hence, there are important limits to the benefits of empathy. It is important to study them to be able to inform physicians and to identify ways to cope with them. We therefore need to temporarily set aside the assumption that what satisfied the descriptive conditions of empathy is necessarily good to explore the effects of the potential descriptive components of empathy more thoroughly. We will then be in a better position to choose a concept of empathy.

### The potential components of empathy

Although there is disagreement on what constitutes empathy in the medical field and in other disciplines, there is a broad agreement that empathy concerns our understanding of others’ feelings and experiences and our own experience of them. The cognitive sciences have identified and studied different processes involved in that wide conception of empathy: perspective-taking, affective empathy, emotional contagion, empathic concern, and empathic distress. We will consider them to be the potential components of empathy.^7^ Surprisingly, literature on physician empathy rarely includes data from the cognitive sciences (exceptions include Preusche and Lamm 2016; Decety 2020). In this part, we describe these processes and report the main empirical findings about them.

Perspective-taking consists in imaginatively taking someone’s perspective. It is often seen as a way to gain a first-person understanding of another’s experience. For example, if we take the perspective of a patient receiving a cancer diagnosis, we might imagine the fear and perhaps the grief that we would feel in that situation as well as what concerns we would have. We can then project them on the patient and, if they are accurate, we have gained a better understanding of their perspective. We can take someone’s perspective by imagining that we are ourselves in their situation (imagine-self perspective) or by imagining that we are this person in their situation (imagine-other perspective). The imagine-other perspective is thought to be more accurate because it takes into account the other person’s character and history (Coplan 2011), although it is not clear that this is the case (Lobchuk and Vorauer 2003; Gouveia et al. 2017). Furthermore, as seen above, we are less good than we think at perspective-taking (Maibom 2018). Hence, the usefulness of perspective-taking in gaining an accurate understanding of another is questionable.

Affective empathy refers to feeling what another person is feeling while remaining aware that it is not our own feelings (de Vignemont and Singer 2006; Maibom 2017). This involves, for example, feeling someone’s grief over a terminal cancer diagnosis. We often feel affective empathy when we take another’s perspective, but we can also feel it spontaneously when witnessing someone suffer or even just by knowing about it (Maibom 2014). For example, when we see a needle entering someone’s hand we automatically cringe (Jackson et al. 2005).

Empathic concern (sympathy in philosophy) is an emotional reaction of concern for another and from the perspective of the “one-caring” (Darwall 1998). This involves feeling worried for someone who is in danger, sad for someone who suffered a loss, etc. (Batson 2011). Batson claims that the two antecedents of empathic concern are valuing a person and perceiving that they are in need (Batson 2011). In other words, we feel empathic concern when we see (or know) that someone we care about is in a situation that we deem bad for them. Empathic concern is experienced as more positive than empathy (Singer and Klimecki 2014; Klimecki et al. 2014) and produces altruistic motivation (Batson 2011).

Emotional contagion consists in feeling what one or several persons are experiencing, but without being aware that we are catching the emotion of others (Darwall 1998; Scheler 2003; Stueber 2010). For instance, a patient might become nervous while in the waiting room because other patients are nervous and might start thinking that they are genuinely feeling nervous themselves. Emotional contagion is often considered the basic mechanism that makes affective empathy possible (Preston and Waal 2002). If one does become aware that the emotion felt is another’s and projects that emotion onto them, one then qualifies as feeling affective empathy (Maibom 2020). The behavioral outcomes of emotional contagion have been less studied than the ones of empathic concern or affective empathy—perhaps because it is more difficult to test due to the lack of awareness of it. Balconi found that emotional contagion with positive emotions led to pro-social behavior (Balconi and Canavesio 2013).

Empathic (or personal) distress is a self-oriented and aversive reaction to another’s suffering (Singer and Klimecki 2014). For example, a physician sees that their patient strongly worries and they start feeling unwell because of it. Empathic distress is not felt for the other, but for oneself and motivates one to withdraw or take care of oneself (Eisenberg and Eggum 2009; Batson et al. 1987; Eisenberg et al. 1989). The inability to bear the suffering of others can also lead to violence. Studies found that parents who feel distressed when their infant cries are more likely of abusing their child (Milner et al. 1995; Perez-Albeniz and de Paul 2003). Hence, unlike empathic concern, empathic distress is not a pro-social attitude and is often considered an unwanted process that should be avoided (Hojat et al. 2005; Thomas et al. 2007; Gleichgerrcht and Decety 2013; Wong 2020). It is however not clear if empathic distress happens only when there is an overarousal (Eisenberg and Eggum 2009) and is always a strong reaction (Singer and Klimecki 2014) or if some degree of personal distress is always present in empathic reactions (Maibom 2020).^8^

These five processes are interconnected. Perspective-taking often leads to affective empathy. When we imagine receiving a cancer diagnosis, and feeling fear and grief, we will often start feeling those emotions. Perspective-taking can also increase empathic concern (Batson 2011). Interestingly, different effects have been found between imagine-other and imagine-self perspective-taking. Unlike the imagine-other perspective, the imagine-self perspective is likely to elicit empathic distress in addition to empathic concern, possibly because we feel more personally involved (Batson 2011; Jackson et al. 2006; Lamm et al. 2007). Because perspective-taking is a process that we can often control and choose to get into, participants in empirical studies are often instructed to take someone’s perspective in order to elicit in them affective empathy or empathic concern (Batson 2011; Sober and Wilson 1999).

Affective empathy, like perspective-taking, can also lead to both empathic concern or empathic distress (Singer and Klimecki 2014). When we feel someone’s suffering, we usually react with concern for that person. However, we can also start feeling distressed (Singer and Klimecki 2014). For example, when sharing the grief and the fear of the patient with a cancer diagnosis, we can feel it so intensely that it becomes overwhelming. The ability to emotionally regulate, i.e. to process difficult emotions and not be overwhelmed by them, reduces the likelihood to feel empathic distress rather than concern (Decety 2020). It is likely that those processes are causally related in other ways that have not been empirically observed yet.

Lastly, we know that empathy is modulated by social factors (Melloni et al. 2014). We tend to feel more affective empathy for people we have close relationships with (Meyer et al. 2013), for those who belong to our in-group and are more similar to us (Stürmer et al. 2006; Tarrant et al. 2009), and those who behave fairly (Singer et al. 2006). Affective empathy is also modulated by attention. When asked to count someone’s fingers, we feel less empathy for the one whose hand is being pricked by a needle (Gu and Han 2007). Similarly, our ability to feel empathic concern depends on whether we like or dislike that person (Batson et al. 2007) and on the number of people in need (Slovic 2010). Hence, we do not feel affective empathy and empathic concern equally for all those who are in need. This is why they have been criticized for being partial and biased (Batson et al. 1995; Bloom 2017).

### Effects of the potential components of empathy on care

Now that we have a good understanding of what the potential components of empathy are, we can explore their effects in a medical interview. Although this description will include empirical findings, it is still speculative.

#### Perspective-taking

As explained above, perspective-taking is considered to help us understand others. A good understanding of the patient is critical to good care since it is needed to make a diagnosis, but also to see what therapeutic path makes sense for the patient and to adapt the way to communicate to them. For example, understanding whether a broken arm is due to a fall or to domestic violence will greatly influence how a physician will address the situation. Being understood is also very important for patients (Wensing et al. 1998). However, perspective-taking is not the best way to gain knowledge about another since we need to know about another’s situation to take their perspective. Physicians primarily learn about patients by observing them, taking their history, doing some tests, etc. Perspective-taking can nevertheless improve patient understanding by helping physicians see what the facts learned means to patients. For example, a fertility professional might freeze and de-freeze embryos frequently and see them as regular organic matter. Patients, however, might see these embryos as their potential future children and it might be very important to them that those embryos are handled in a particular way. Taking the patient’s perspective might then help the physician to be mindful of that.

However, as we have seen, perspective-taking does not guarantee accuracy. If a physician’s perspective-taking is based on false information, it will be of no help. For instance, a gynecologist might assume that their patient with PCOS (polycystic ovary syndrome) is worried about their fertility and imagine what it must be like for them, while the patient has no plan to conceive. Furthermore, as we have seen, it seems that we are not proficient at imagining how we would react in a given situation if we were someone else (Maibom 2018). When the result of perspective-taking is inaccurate, it can lead to inappropriate care and the patient might stop trusting their physician and feel misunderstood and isolated.

#### Affective empathy

Affective empathy can also help with understanding patients. Spontaneous affective empathy can help physicians identify their patient’s emotion(s). For example, feeling the patient’s shame over having been sexually assaulted might help the physician care for that patient. Like perspective taking, affective empathy can be inaccurate and have negative consequences. For example, if the patient is not feeling shame but anxiety of being assaulted again, the shame that the physician thinks they are sharing with the patient will mislead them.

A longstanding worry with affective empathy is that it might threaten physicians’ objectivity and wellbeing. If a physician feels the pain and grief of a patient with terminal cancer intensely, their emotional state might make them unable to view the situation with the distance necessary to discern the best option for the patient. The physician might then decide that everything should be tried to save the patient despite the very negative odds and spend valuable resources on exploring options that are only painful for the patient. Feeling strong negative emotions repetitively might also lead to personal distress if one is not able to self-regulate, which in the long term can lead to compassion fatigue (Gleichgerrcht and Decety 2013, 2014). Furthermore, it is not helpful for a patient to see that their physician is in the same negative emotional state as they are. A patient who just learned that they are ill need reassurance and support rather than more anxiety or grief (Jamison 2014). Nevertheless, short spans of affective empathy might still be beneficial for physicians to understand how the patient is feeling.

#### Empathic concern

Feelings of empathic concern for a patient indicate that the physician value them. If the patient perceive it, they can feel cared about and supported, which is important to them (Bensing et al. 2013). Empathic concern can thus contribute to a good doctor-patient relationship and improve healing outcomes (Gleichgerrcht and Decety 2014). Furthermore, since empathic concern motivates altruistic behavior, the physician feeling empathic concern will feel motivated to help their patient (Batson 2011). This might contribute to motivating the physician to investigate and find the best course of action for the patient.

However, empathic concern can also potentially have a negative impact on care. If empathic concern always involves a component of emotional distress, it might be difficult for physicians to experience empathic concern regularly. If, on the opposite, empathic concern is experienced as a positive state (Singer and Klimecki 2014), feeling empathic concern might benefit physicians. Gleichgerrcht and Decety found that physicians who feel empathic concern are more satisfied with their job and have a better quality of life (Gleichgerrcht and Decety 2013, 2014). Too much empathic concern might also cause distress in physicians when they are unable to help. In addition, physicians that care a lot about their patients might be determined to help them even if it becomes futile.

Lastly, because of their empathic concern, a physician might be motivated to help their patients in a way that is harmful for others or society at large (Batson et al. 1995; Bloom 2017). For instance, a physician might prescribe a treatment that is far too expensive given its effectiveness.

#### Emotional contagion

If a physician experiences emotional contagion, they will feel an emotion that they consider theirs. If they become aware that they are experiencing emotional contagion, their emotional episode can transform into affective empathy and contribute to patient understanding. This is what happened in Halper’s example with her hopelessness about the successful executive (see “Introduction” section) (Halpern 2001). By contrast, if there is no awareness of emotional contagion, it will not help them understand the patient. Emotional contagion can then prevent a physician from feeling affective empathy and empathic concern. Furthermore, if the emotion felt has a negative valence (fear, sadness, grief, etc.), the physician experiencing emotional contagion might not feel well and need to process that feeling.

#### Empathic distress

There does not seem to be any direct benefit for a physician to feel empathic distress. If a physician feels distressed and needs to take care of themselves, they do not have the mental and emotional resources to listen to the patient, make a diagnosis and show support. Empathic distress also prevents physicians from feeling empathic concern. If such episodes last or happen frequently, it might cause emotional fatigue and impact the quality of life of the physician, even leading to burnout (Gleichgerrcht and Decety 2013).

#### Empathy during the medical interview

The potential components of empathy can thus have positive or negative effects depending on how they are instantiated (see Table 1). Furthermore, their impact also depends on when they occur during the interview. There are times when these empathic components will be very important to understand the patient and develop a good relationship with them. Showing understanding might be especially important during history taking and when the patient is talking about their life, how they feel, their symptoms, etc. The expression of support and reassurance (empathic concern) might be especially important before a painful test or at the end of the interview. At other times, these processes might negatively impact care. For example, affective empathy while performing a lumbar puncture might distract the physician and raise the chances of harming the patient.

**Table 1:** Table that summarizes the positive and negative effects that the components of empathy can have on care

Component of empathy	Potential positive effects	Potential negative effects
Perspective-taking	• (Experiential) understanding of the patient	• Inaccurate understanding of the patient
Affective empathy	• Affective understanding of the patient	• Inaccurate understanding of the patient
		• Lack of objectivity
		• Too emotionally demanding for the physician/compassion fatigue
Empathic concern	• Motivation to help	• Ethical risks of wanting to help too much
	• Patient feels supported and cared for	• Too much personal involvement
Emotional contagion	• Understanding of the patient (if awareness of emotional contagion)	• Negative feelings/distress
		• Hinders affective empathy and empathic concern
Empathic distress		• Emotionally distressing
		• Needing to take care of oneself rather than of the patient
		• Hinders empathic concern

However, this does not imply that components of empathy should be instantiated only at clearly dedicated moments such as history taking or at the end of consultation. The expression of concern and support might be needed during an intervention that makes the patient anxious or mid-clinical thinking to make sure it is based on accurate information. The patient might also add some new information at the end of the interview, making perspective-taking and affective empathy useful. Furthermore, although we have been focusing on the what the physician can do, a medical interview is an interactive process in which the patient plays an active role and collaborates with the physician to reach an understanding of the issue and agree on a path forward. The usefulness of the potential components of empathy depends on that collaboration. The practice of the different components of empathy for good care is therefore a complex and fluid enterprise that requires a lot of know-how and, therefore, experience.

#### The three roles of empathy

It appears that the five processes can contribute to good care in three main ways. First, perspective-taking, affective empathy and, to a certain extent, emotional contagion, can help physicians understand their patients. Second, when communicated to patients, verbally or non-verbally, most of these components can contribute to a good doctor-patient relationship. Perspective-taking, affective empathy and emotional contagion can make patients feel understood; empathic concern can make patients feel cared for, valued, and supported. This can encourage patients to confide further in their physician, can help with the building of a therapeutic alliance and improve healing outcomes (Neumann et al. 2011). Lastly, empathic concern can contribute to good care by motivating physicians to help the patients.These effects correspond to the three roles that have been (implicitly) endowed to empathy, namely, epistemic (understanding the patient), relational, and motivational (Neumann et al. 2007).

However, as we have seen, all the potential components of empathy can also impact care negatively. Furthermore, these processes are not sufficient to fulfill the three roles of empathy. Perspective taking, affective empathy and emotional contagion are not sufficient to gain a good understanding of the patient. Similarly, developing a good relationship with the patient will also require other relational qualities such as kindness, politeness, humor, etc. Lastly, while empathic concern is sufficient to motivate physicians to help patients, other motivations are available to them. Physicians can also be motivated to help patients because of a sense of professional duty, for instance. These other motivations are especially important when a physician does not feel empathic concern, because the patient is rude or annoying. Hence, none of the processes of empathy guarantees good care.

### For a descriptive and encompassing conception of empathy

The above description suggests that there is no component that only produces positive effects. This implies that there is no definition of empathy such that what satisfies its descriptive conditions is necessarily good. Therefore, the idealization of empathy puts physicians at risk by ignoring its potential negative effects.

Furthermore, we argue that we should adopt a purely descriptive definition of empathy. Since there is no ideal descriptive component of empathy, for any definition of empathy as a thick concept, there will be cases where physicians satisfy the descriptive conditions, but not the normative ones. Physicians would then not qualify as empathic. For instance, the gynecologist who assumes that their patient is afraid of labor pain might be said empathic if they are correct, but not if the patient is in fact afraid of losing control. This would imply that physicians cannot assess whether they are empathic or not. This disjunctive view makes the concept of empathy overly complex and confusing and might make it harder for physicians to achieve mastery of empathy. Conceiving of empathy as purely descriptive implies, however, that we could qualify a physician as empathic even when it does not yield benefits. Although some might find this counterintuitive, it might help to convey a more nuanced view of empathy. Furthermore, empathy can still have a positive connotation and be considered positive and helpful overall.

We also argue that our conception of physician empathy should be wide and all-encompassing. Restricting empathy to perspective-taking or empathic concern to encourage physicians to focus on them might be appealing to some. However, since the potential components of empathy are interrelated, defining empathy as perspective taking or empathic concern will not prevent physicians from feeling affective empathy or empathic distress. A wide conception of empathy that refers to the five processes discussed would have the advantage of leaving all idealization behind and helping physicians understand their experience. Understanding the risks and benefits of all the processes would also help them identify which form of empathy is needed depending on the situation and their competences. Empathy would not be presented as an ideal to reach, but as a complex tool that they need to learn and practice to master. Furthermore, we can help physicians master empathy by teaching them competencies and strategies that will help them use empathy in a way that is most beneficial for them and their patients.

#### Teaching and training empathy

Empathy is often taught with communications training, but also through medical humanities (fiction, poetry, theater), and other interventions, such as role-playing, patient-shadowing, reading and writing narratives (Patel et al. 2019; Hojat 2009). Learning from role-models also appears to be an important way in which medical students learn about empathy (Krishnasamy et al. 2019; Seeberger et al. 2020; Thangarasu et al. 2021). All those interventions aim at helping medical students take the perspective of their patients, understand them, and communicate and behave in a way that make patients perceive them as empathic and compassionate (Hojat 2009). Reviews of literature suggest that such interventions are effective at enhancing empathy levels in physicians and medical students (Stepien and Baernstein 2006; Batt-Rawden et al. 2013; Kelm et al. 2014; Patel et al. 2019). However, this literature faces the same difficulties as the one on the effects of empathy. Furthermore, few are randomized controlled trials and few measure long-term impact. Furthermore, although we have studies evaluating specific empathy training programs, we know little about how empathy is taught in medical schools in general. It appears that the competences that are most often trained are communication skills and perspective-taking skills although others are likely trained as well (Stepien and Baernstein 2006; Batt-Rawden et al. 2013).

The neutral conception of empathy that we have developed suggests that other competences and strategies could be trained as well to support physicians in their practice of empathy. Medicine students would benefit from learning strategies to avoid the negative effects of empathy and promote the positive ones and strategies to cope with the negative effects if they occur.

An important ability to develop for an optimal use of empathy is emotion regulation. Emotion regulation is “the ability to respond to the ongoing demands of an emotional experience in a manner that is socially tolerable and sufficiently flexible to permit spontaneous reactions” (Decety 2020). Those who have difficulty with emotional regulation are likely more at risk of feeling personal distress (Powell 2018). Emotional regulation involves strategies (e.g., avoiding the emotion eliciting object) and abilities (e.g., emotional awareness) (Weilenmann et al. 2018). More research is needed to determine what the best strategies and abilities to teach to physicians and how to do so.

Several specific strategies can also be used to optimize the use of empathy. For instance, physicians could learn to use the imagine-other rather than an imagine-self perspective (Lamm et al. 2007). This would lower the chances of physicians experiencing empathic distress and would help them think about what the patient, with their history, character traits, preferences, etc. experience rather than what they would experience in the patient’s situation.

To improve patient understanding, it would be helpful for physicians to take into account their own biases (FitzGerald and Hurst 2017). They can become aware of those through implicit association tests, for instance, and keep them in mind when dealing with patients who belong to groups they are biased against. To avoid misunderstanding, physicians need to remember that their understanding can be wrong (Nakar et al. 2007; Quirt et al. 1997) and should develop the habit of sharing their understanding to the patient and ask if it is accurate. Furthermore, if they see that affective empathy or empathic concern would be helpful but they are not spontaneously feeling it, they can actively try to take the patient’s perspective to try to elicit those feelings (Sober and Wilson 1999).

Some strategies also need to be developed at an institutional level to deal with cases where empathy goes wrong. For example, a hospital might offer support by providing a professional who can listen and support physicians who are distressed, by offering mindfulness or resiliency training, etc. (Rosenstein 2019). Similarly, a strategy needs to be developed for when ethics has been breached. Here again, institutional policies will be important. For example, a hospital might have a policy of transparency that encourages physicians to come forward when such cases have happened and offer support to find a solution with the stakeholders and if possible, repair the harm done (‘Creating an Ethical Culture Within the Healthcare Organization’ n.d.).

## Conclusion

In this paper, we have argued that the idealization of empathy has made a consensus on a definition for empathy hard to achieve and has prevented us from gaining a precise understanding of the effects of the different components of empathy. We proceeded to fill that gap by introducing the potential components of empathy as they have been identified in the cognitive sciences and explored their effects in the context of the medical interview. That description showed that all the potential components of empathy (perspective-taking, affective empathy, emotional contagion, empathic concern, and empathic distress) can have negative effects depending on how they are instantiated and when in the medical interview. This suggests that there is no ideal concept of empathy. Since bypassing the negative effects of empathy prevents physicians from knowing about them and learning how to avoid them, we argued that empathy in medicine should be conceived as encompassing all the potential components of empathy and as leading to both positive and negative effects on care. Lastly, we outlined some competences and strategies that can be used to minimize the risks of empathy. Further empirical work is needed to verify our hypotheses concerning the effects of the different components of empathy and to identify the best strategies to manage the risks of empathy.

## Footnotes

1 A recent meta-analysis suggests that the finding of a significant difference in empathy levels between third and first year medical students depends on the empathy scale used (Spatoula et al. 2019).

2 It is not clear when this concept that originated in aesthetics became evaluative.

3 This raises important philosophical issues regarding the “is-ought gap”. See (Blomberg 2010; Väyrynen 2021).

4 This line of reasoning that defines empathy according to what attitude or behavior is needed from the physician to have a certain effect on the patient is probably why some empathy tests also measure interpersonal skills that are not directly related to what we commonly call “empathy” like having a sense of humor (JSEP) and being positive (CARE) (Hojat et al. 2018; Mercer and Reynolds 2002).

5 Exceptions include for example Gleichgerrcht and Decety 2014 and Kim et al. 2004.

6 By contrast, the Interpersonal Reactivity Inventory provides four subscales (Davis 1980; 1983).

7 We do not claim that this list is exhaustive. However, we exclude processes that are not related to the wide understanding of empathy such as a sense of humor or helping behavior, which is usually considered an outcome of empathic concern (see below).

8 While personal distress and empathic concern are felt only when another person is perceived as in need, the other processes can also occur when the other is well.
